# Supplementary material for: Are carbon emissions trading and green financial instruments synergistic? -Comprehensive quantitative research based on content analysis
Source: PLoS One. 2024 Mar 7;19(3):e0298601. doi: 10.1371/journal.pone.0298601 (PMC10919601; doi:10.1371/journal.pone.0298601)
Supplement: S1 Data — (ZIP) [file pone.0298601.s001.zip › Data/地级市Z_F工作报告.zip!/╜¡╬≈/╙Ñ╠╢╩╨/└┤╫╘╣1⁄2╓┌║┼í╛┬φ┐╦╩2╛▌═°í┐_2006.docx]

各位代表：

现在，我代表市人民政府，向大会作政府工作报告，并就《鹰潭市国民经济和社会发展第十一个五年规划纲要（草案）》作出说明，请予审议，并请市政协各位委员和列席同志提出意见。

一、关于“十五” 回顾和二00五年政府工作

“十五”时期，是鹰潭经济社会发展不平凡的五年。五年来，全市人民在省委、省政府和市委的正确领导下，坚持以邓小平理论和“三个代表”重要思想为指导，树立和落实科学发展观，解放思想、开拓创新，同心同德、善谋实干，战胜了前进道路上的一个又一个困难和挑战，全面超额完成了“十五”时期的各项任务，进一步开创了我市经济社会发展和改革开放的新局面。主要表现在：

──综合经济实力迈上新台阶。全市生产总值由2000年的53.95亿元增加到2005年（下同）的118.62亿元，按可比价格计算，年均增长14.6%，比“十五”计划目标超过35.62亿元。按现行汇率换算，人均生产总值达到1385美元。财政总收入由2000年的5.11亿元增加到14.44 亿元，年均增长23.1%，比“十五”计划目标超过6.94亿元，人均财政总收入达1325元；其中地方财政收入由2000年的2.99亿元增加到6.71亿元，年均增长17.55%，比“十五”计划目标超过2.31亿元。贵溪市财政总收入突破10亿元，余江县财政总收入突破1亿元。

──经济结构调整取得新进展。三次产业结构由2000年的20.1：41.1：38.8调整到14.5:49.7:35.8，五年内二产上升8.6个百分点，呈现出工业化程度提高的阶段性特征。工业园区从无到有，投入逐年加大，发展势头迅猛，一批企业投产见效，成为新的经济增长极。全市规模以上工业增加值年均增长17.6%，提前一年实现在2000年基础上翻番的目标任务。农业基础地位更加巩固，抵御了洪水、干旱和禽流感等多种灾害，粮食生产连年丰收。粮食总产量由2000年的48.16万吨增加到55.60万吨。旅游经济进一步发展，现代流通体系不断完善。

──城市面貌发生历史性变化。坚持以规划为龙头，聘请国内知名设计单位对鹰潭市城市总体规划进行修编，对城市发展进行科学定位。城市基础设施建设规模空前，新建了龙虎山大道、沿江大道、天洁路、梨温高速公路鹰东连接线等城市骨干道路，突破了铁路和信江对城市发展的制约，拉大了城市框架。市区建成区面积由2000年的12平方公里扩大到22.1平方公里。打造了一批市政精品工程，建设了东湖公园等一批市民休闲场所。对交通路、胜利路、四海路、湖西路和梅园大道进行了全面升级改造。新建了一批高品位的住宅小区。鹰潭新汽车站建成投入使用，出租车全部更新换型。开展了一系列旨在提高市民素质的活动，城市文明程度提高，城市面貌焕然一新，品位大幅提升。贵溪市、余江县城市面貌也发生了巨大变化，聚集和辐射功能明显增强。全市城市化率由2000年的34.9%提高到2005年的41.8%，五年内提高了6.9个百分点。

──改革开放取得新突破。各项改革整体推进。农村税费改革不断深入，林业产权制度改革进展顺利。市县乡三级机构改革全面完成，事业单位人事制度改革稳步推进。卫生体制改革初见成效，公共卫生体系建设步伐加快，非典、人感染禽流感等重大疾病防治取得阶段性成果，新型农村合作医疗试点进展顺利。粮食流通体制和市场化改革步伐加快，粮食收购市场进一步规范。企业产权制度改革取得阶段性成效。市磷肥厂政策性破产已获正式批复，依法处置市直部分改制企业历史债务约3亿元。财政体制改革取得新进展，部门预算制度全面实施，政府集中采购制度日趋规范，乡财县代管试点工作正在积极开展。

开放型经济快速发展。坚持实施大开放战略，对接“长珠闽”，加速融入全球化进程，不断提升对外开放水平。实际引进市外资金由2000年的5.44亿元增加到26.0亿元，年均增长36.7%；实际利用外资由2000年的529万美元增加到5600万美元，年均增长60.3%。外贸出口由2000年的661万美元增加到3247万美元，年均增长37.5%。

──经济社会发展后劲明显增强。全社会固定资产投资由2000年的11.04亿元增加到60.1亿元，年均增长40.3%，比“十五”计划目标超过40.7亿元。全市各项贷款余额由2000年的72.16亿元增加到110.34亿元；各项存款余额由2000年的57.52亿元增加到123.69亿元，增长1.2倍。

──科技教育及社会事业成绩显著。科技创新能力得到提升，科技成果转化加快。教育事业发展加快，办学条件明显改善，完成了鹰潭职业技术学院的组建。计划生育工作继续加强，人口增长得到有效控制。医疗保健能力进一步提高，公共卫生体系建设得到加强。文化、广播、电视、新闻、出版、体育等社会事业也都有新的进步。

──人民群众生活水平稳步提高。全市社会消费品零售总额由2000年的19.11亿元增加到36.41亿元，年均增长13.8%。全市城镇居民人均可支配收入由2000年的5342元增加到8843元，年均增长10.6%，比“十五”计划目标超过1523元。农民人均纯收入由2000年的2380元增加到3640元，年均增长8.9%，比“十五”计划目标超过530元。全市居民城乡储蓄存款由2000年的39.12亿元增加到82.46亿元，增长1.1倍。

──依法治市进程不断加快。认真实施《行政许可法》，不断规范行政许可行为。市本级行政审批事项由964项清理减少到319项，行政许可实施主体明确为45个。落实社会治安综合治理领导责任制，加强了社会治安防控体系建设，2003年至2005年连续三年被评为全省社会治安综合治理目标管理先进市。各项安全生产工作进一步加强，安全生产形势平稳。“四五”普法顺利通过省验收。反腐倡廉工作深入推进，损害群众利益的突出问题得到有效治理，发展环境明显优化。建立专家咨询、市民介入的决策机制，努力做到科学民主决策。

各位代表，2005年是实施“十五”计划的最后一年，也是超额完成“十五”计划的关键一年。这一年，我们深入贯彻党的十六大和十六届三中、四中、五中全会精神，紧紧围绕“建设和谐平安鹰潭、共创富民兴市大业”主题，一手抓保持共产党员先进性教育活动，一手抓经济社会发展，全市经济社会发展取得了新的可喜成绩。全市生产总值比上年（下同）增长16.4%；财政总收入增长35.4%，其中地方财政收入增长26.7%；全社会固定资产投资增长36.8%；社会消费品零售总额增长16.6%；城镇居民人均可支配收入增长14.5%，农民人均纯收入增长11.5%。主要经济指标增幅均高于全省平均水平。一年来，我们主要做了以下几方面的工作：

1、“三农”政策全面落实，农业综合生产能力不断提高。开展了为期三个月的千名干部下乡为民办实事活动，全面落实了一系列促进粮食增产、农民增收的政策措施。全面取消了农业税，实现了合同内农民零负担目标。对农民实行了粮食直补、良种补贴和农机具购置补贴。全市免征农业税4100万元，发放粮食直补、良种补贴和农机具购置补贴资金2738万元，及时启动了早、中、晚稻托市收购预案，认真落实最低收购价政策，保护了农民的利益。加快推进农业产业化，培育了一批省、市级农业产业化龙头企业和合作经济组织，规模以上农业产业化龙头企业销售收入增长18.6%。开展了送科技、文化、法律三下乡活动，加大了农村劳动力培训力度。组织实施了退耕还林、国债长防林、生态公益林、农发长防林等一批林业重点工程，森林资源稳步增长，生态环境明显改善。实施了余江白塔渠挖潜改造工程、血防水利环治工程和中潢圩堤、硬石岭水库、塘湾水库等一批病险水库除险加固工程，组织了农业综合开发项目，有效地改善了农业和农村生产、生活条件。实施了土地利用总体规划，耕地保护、土地市场管理新机制进一步健全和完善。社会主义新农村建设起步良好，全市已有58个自然村开展了社会主义新农村建设试点，30%的自然村已经制定了新农村建设规划。100%的乡镇通了油（水泥）路，99.5%的行政村通了公路，71%的行政村通了水泥路。

2、工业化进程快速推进，工业经济效益进一步提升。全市规模以上工业企业实现工业增加值54.50亿元，增长24.6%；实现利税总额34.84亿元，增长62.7%。规模以上工业经济效益综合指数226.3%,同比提高16.4个百分点。工业经济各项主要指标在全省排位靠前。重点企业发展迅猛。江西铜业集团公司年销售收入超200亿元，成为全省和中国铜工业第一家销售收入超200亿元的企业，新上30万吨铜冶炼项目已正式开工。贵溪电厂扩建两台30万千瓦机组两年内建成投产，总装机达110万千瓦。黄金埠电厂锦江50万千伏开关站已开工建设。一批铜材加工、眼镜、微型元件企业相继投产，水表、节能灯、制药、木雕等特色产品快速增长。全市工业“十一五”专项规划和商业网点规划已全面启动。工业园区基础设施建设初具规模，聚集效应凸显。全市园区落户企业达161家，投产企业102家，实现利税2.07亿元，安置就业1.39万人。

3、旅游经济稳步发展，第三产业不断壮大。举办了“鹰潭（龙虎山）旅游发展恳谈会”，编制了全市“十一五”旅游发展规划，强化了发展旅游经济的共识共举。成功举办了2005中国（鹰潭）道教文化节暨第九届龙虎山道教文化旅游节、首届海峡两岸道教文化论坛、天师府建府九百周年庆典等系列活动，参加了省政府主办的2005中国红色旅游博览会，提高了鹰潭和龙虎山的知名度。举全市之力，创建中国优秀旅游城市取得圆满成功。龙虎山风景名胜区被列入首批中国国家自然与文化遗产预备名录。完善了一批景点，建设了一批基础设施，景区和市区旅游接待能力有了新的提高。全市接待国内旅游者507万人次，增长12.6%；接待境外旅游者3.5万人次，增长14.4%；旅游总收入11.8亿元，增长34%；旅游创汇580万美元，增长13.7%。

支持和引导个体私营等非公有制经济加快发展，拓宽了民资准入领域，降低了准入门槛。全市个体户户数、从业人员、注册资金分别比上年增长10%、9%和31%，私营企业户数、从业人员、注册资金分别比上年增长16%、33%和26%。

4、城市化进程明显加快，规划、建设和管理水平迈上新台阶。编制完成《鹰潭市市域城镇体系规划》和城市专项规划。开展创建省级卫生城市活动通过了省考核鉴定。创建省园林城市、全国双拥模范城市工作力度不断加大。市区环境卫生状况大为改善，城市形象得到明显提升。城市基础设施建设顺利推进。江南水厂扩建、管道燃气、污水处理、体育中心、市人民医院门诊医技大楼等工程建设正在抓紧实施。垃圾填埋场一期、消防指挥中心等一批重点工程顺利完工，院里路、51号路竣工通车，胜利休闲广场、东湖二期、月牙湖、供水管网等一批改造工程施工进度加快。市政府与国家开发银行江西分行签订了2.9亿元城市建设项目贷款协议，用于建设雄鹰大道、夏埠沿江大道、龙虎山大道延伸段等一批骨架工程。贵溪市、余江县也分别与国家开发银行江西分行签订了1.9亿元、2000万元的城市建设项目贷款。加快了7个省级以上重点小城镇建设，以推进城乡一体化进程。城市管理体制进一步理顺，“两级政府、三级网络、四级管理”的城市管理格局开始形成。

5、招商引资取得实效，对外开放水平进一步提升。按照省委、省政府决策部署，认真开展了“重大项目招商年”和“诚信优质服务年”活动。进一步健全了客商服务体系，完善了政务环境评议评价制度，建立了行政投诉中心，提升了服务水平，我市被浙江省评为“2005浙商最佳投资城市”。突出了专业、项目、产业、区域招商，依托铜、眼镜等特色产业，着力引进了一批关联度强的工业配套项目。实际引进5000万元以上省外工业项目资金23.19亿元，增长85.0%；实际利用外资增长56.5%，外贸出口增长33.6%。我市被省政府评为引进省外资金先进设区市。

6、各项社会事业统筹发展，精神文明建设深入人心。坚持科教兴市，开展了与天津大学的全面合作。铜产业发展规划已通过专家评审。教育投入加大。全市共投入中小学危房改造资金1478万元，改造危房4.2万平方米，基本消灭了现存D类危房。优质高中建设进展顺利。义务教育阶段实行“两免一补”受益学生2.4万人。职业教育取得较大发展，一所学校被评为国家级重点中专，两所学校被评为省级重点中专。卫生事业继续发展，市和贵溪、余江疾控机构建设项目均已竣工，余江县新纳入新型农村合作医疗试点县，重大疾病防治和社区卫生服务网络建设进一步加强。计划生育基层基础工作进一步巩固，整治性别比例失调专项工作取得成效，人口自然增长率7.35‰，控制在省下达目标之内。积极参加第三届江西艺术节，创作了一批文艺精品。新体育中心的市体育馆已开工建设，竞技体育取得了新突破，全民健身活动连续十年获得国家体育总局表彰。广电网络建设规模不断扩大，农村“村村通”工程建设步伐加快。全市第一次经济普查获得成功。精神文明建设进一步加强，多项工作和一批个人荣获全国、全省先进称号。在全市范围内认真开展了保持共产党员先进性教育活动和全民创业主题教育活动，市民素质进一步提高，创业热情空前高涨。召开了全市劳动模范和先进工作者表彰大会，全市涌现出一批省、市劳动模范。余江县锦江镇九亭村被司法部、民政部授予“全国民主法治示范村”。施华山同志被公安部授予全国公安系统一级英雄模范称号，成为全市党员干部和群众学习的楷模。审计、人事编制、档案、环境保护、人防、民族宗教、外事侨务、气象服务、防震减灾、民兵预备役、老龄、残疾人等各项事业健康发展。

7、社会保障体系不断完善，社会稳定和安全生产形势明显好转。坚持每个季度举办一次大型就业招聘洽谈会，落实就业再就业各项政策。全市新增城镇就业人员2.1万人，下岗失业人员再就业5632人，困难群体再就业1512人，发放小额担保贷款2054万元。落实了“三条保障线”制度，全面完成了省政府下达的扩大就业和社会保险目标任务。调整了全市企业离退休人员养老金标准，城市居民最低生活保障标准由原每人每月120元调增为160元。稳步实施了农村特困户救济救助和农村五保“敬老工程”，开展了一系列扶贫济困送温暖活动。认真贯彻落实新《信访条例》，畅通信访渠道，规范信访工作程序，解决了一批信访疑难问题。组建了市公安特警支队，集中开展了一系列“严打”整治斗争、矛盾纠纷排查调处工作，维护了社会的稳定。出台了一系列突发性公共事件应急预案，确保了突发性公共事件处理工作有章可循。积极建立和完善了食品和药品监管“两个体系”，食品药品市场不断规范。加强了生产安全、交通安全、防火安全和食品安全工作，有效预防和减少了一般伤亡事故，杜绝了重特大安全责任事故的发生。

回顾过去的工作，我们清醒地看到，经济社会生活中还存在不少困难和问题，主要是：经济总量仍然偏小，地方企业规模有待进一步做大；第三产业比重不高，区位优势和资源优势有待进一步挖掘；就业和再就业压力仍然较大，解决“三农”问题任务很重；影响社会和谐稳定的因素不少，政府依法行政的能力有待进一步提高。对这些问题我们将高度重视，并用改革和发展的办法，努力克服和解决。

各位代表，“十五”期间和2005年取得的成绩，是全市人民在市委的正确领导下，认真贯彻落实党中央、国务院和省委、省政府的大政方针，团结奋斗，扎实工作的结果，是市人大依法监督和市政协民主监督、支持、参与的结果，是各民主党派、工商联、无党派人士、各人民团体和驻鹰单位、人民解放军、武警官兵以及离退休老同志共同努力和大力支持的结果。在此，我代表市人民政府，向全市人民，向所有关心、支持我市改革与发展的同志们，表示衷心的感谢并致以崇高的敬意！

二、关于“十一五”规划

“十一五”时期是全面建设小康社会的关键时期，也是实现鹰潭加速崛起的关键时期。我们必须进一步增强历史责任感和时代紧迫感，紧紧抓住和用好重要战略机遇期，努力工作，应对挑战，认真解决经济建设和社会发展中存在的矛盾和问题，突破发展的瓶颈制约和体制障碍，努力开创我市经济建设、政治建设、文化建设、社会建设的新局面。下面，我就《鹰潭市国民经济和社会发展第十一个五年规划纲要（草案）》作如下说明。

根据《中共鹰潭市委关于制定全市国民经济和社会发展第十一个五年规划的建议》，市政府制定了《鹰潭市国民经济和社会发展第十一个五年规划纲要（草案）》（以下简称《纲要（草案）》）。《纲要（草案）》坚持以科学发展观统领经济社会发展全局，着眼鹰潭加速崛起和富民兴市，总结过去，展望未来，在分析经济社会发展现有条件和面临的新形势基础上，提出了我市“十一五”时期经济社会发展的总体要求、奋斗目标和主要任务。

“十一五”时期经济和社会发展的总体要求是：以邓小平理论和“三个代表”重要思想为指导，全面落实科学发展观，坚持以大开放为主战略，以招商引资为主抓手，以转变经济增长方式为突破口，积极顺应推进农业农村现代化、新型工业化、新型城镇化、经济国际化和市场化大趋势，按照“扬优成势、以特取胜”的理念，突出重点，协调发展，以更大的力度做强工业，做旺旅游，做活商贸，提升城市集聚辐射能力，建设社会主义新农村，在推动全民创业、构建和谐社会进程中，加快由区划小市向经济强市的历史性跨越，为实现鹰潭加速崛起和全面建设小康社会的宏伟目标打下更为坚实的基础。

今后五年经济社会发展的主要目标：一是经济实力进一步增强。按可比价计算，全市生产总值年均增长13%，人均生产总值达到2500美元。财政总收入年均增长15%。固定资产投资年均增长21%。社会消费品零售总额年均增长14%。居民消费价格上涨幅度年均控制在3%左右。二是经济增长质量明显改善。资源利用效率显著提高，万元生产总值能耗比“十五”期末降低20%。自主创新能力增强，科研成果向现实生产力转化加快，科技进步对经济增长的贡献率达到50%以上。三是经济结构继续优化。三次产业结构调整为9.7:52.5:37.8。农业综合生产能力进一步提高，工业在经济总量中的比重达到50%以上，服务业层次上升。四是人民群众生活水平和生活质量提高。到2010年，城镇居民人均可支配收入超13600元，农民人均纯收入超5100元。农村公共服务设施大为改善，社会和居民小区文化设施建设进一步加强。就业岗位持续增加，社会保障体系比较健全。继续稳定低生育水平，科教文卫体等方面条件大为改善。五是生态环境得到有效保护。到2010年，全市森林覆盖率达到57%，城市绿化覆盖率达到35%。主要污染物排放大幅度减少，城乡环境质量得到进一步改善，城市生活污水集中处理率超过70%，生活垃圾无害化处理率达到85%，城市环境质量继续保持国家环境保护模范城市的标准。实现上述目标，必须大力抓好以下几项工作：

第一，大力推进农业农村现代化，建设社会主义新农村。《纲要（草案）》提出，“十一五”期间要贯彻工业反哺农业、城市支持农村的方针，按照“生产发展、生活宽裕、乡风文明、村容整洁、管理民主”的要求，统筹规划好新农村建设，着力提高农业综合生产能力，走出一条以工促农、以城带乡的城乡共同发展新路子。巩固提高粮食生产能力，保护基本农田，完善基础设施，使粮食总产量稳定在50万吨左右。优化农业生产结构，以工业理念发展特色种植业、养殖业，力争到2010年，全市经济作物产值占种植业的比重达65%以上，养殖业产值占农业总产值的比重达65%以上。推进农业产业化经营，大力发展龙头企业，完善龙头企业与农户利益联结机制。发展多种形式的农村合作经济组织，提高农业生产的组织程度。加强农村生态环境和农业基础设施建设。到2010年，全面完成余江白塔渠水利血防综合治理巩固工程等一批水利设施建设，加快园田化、土地整理、农村沼气、农田林网、林业重点生态工程建设，继续实施好贵溪、余江国家沼气能源建设国债项目。加快农村公路建设，力争到2007年底全市100%的行政村通水泥路。加快发展农村社会事业，改善农村生态环境，提高农村农民健康保障、环境卫生、文化教育和精神文明水平。

第二，加快产业结构优化升级，加速推进新型工业化。做强主导产业。力争“十一五”期末，铜冶炼能力达70万吨，铜产业销售收入达500亿元，成为全国最大的铜冶炼及铜加工基地、铜技术开发中心和铜产品集散中心；力争尽快将眼镜加工区、微型元件生产基地发展成为全省乃至全国具有重要影响力的生产制造基地之一；力争“十一五”期末重点企业数量增加、规模扩大、销售收入提高。优化产业结构，大力推进自主创新和技术进步。拓展发展载体。做大市工业园、贵溪工业园、余江工业园，建设320国道工业带，使其成为产业集群比较发达，技术和资金相对密集，对全市发展起主导作用的工业带，成为海外及沿海发达地区产业转移的承接基地。

第三，统筹城乡发展，加速推进城市化进程。强化“大鹰潭”发展理念，拓展城市发展空间。按照“一个核心区、三个特色区”的构想，通过“东连、西接、南扩、北拓”加快城市发展。修建鹰潭至贵溪的城际快速干道，完成鹰潭至贵溪的320国道拓宽改造工程，完成胜利西路延伸至206国道工程，改造320国道至景鹰高速公路接口段，完成龙虎山大道延伸工程，加快开发宝山片区，完成夏埠沿江大道建设，力争2010年主城区建成区面积达到30平方公里。全市城市化率达48%。完善城市功能。优先发展城市公共交通，加强城市供水能力建设。“十一五”期间新增城市供水能力10万吨，城市自来水普及率达到100%，县城自来水普及率达到95%。加强城市环保设施建设。建成市区生活垃圾填埋场、生活污水处理厂。着力改善城市环境，建设规模合理的城市绿地。按照“因地制宜，扬优成势”的发展思路，做大做强县域经济。统筹城乡发展，加快发展小城镇。

第四，加快改革开放，增强发展动力与活力。努力提高外向型经济工作水平，“十一五”期间，力争吸收外商直接投资年均增长15%，外贸出口年均增长25%。优化开放环境。进一步改进政府服务，提高办事效率。全力营造高效、安全的投资环境和诚信鹰潭品牌。力争2007年前在我市设立进出口检验检疫分支机构，2009年前设立海关分支机构。加大改革力度。深化国有企业改革，完善国有资产监督、管理和运营体制，防止国有资产流失。切实落实鼓励、支持非公有制经济发展的政策措施，提高非公有制经济在经济总量中的比重。进一步推进行政管理体制改革，分类推进事业单位改革。

第五，以旅游商贸为龙头，加快现代服务业发展。坚持大旅游发展战略，努力构建“以市区为主体，龙虎山景区为龙头，贵溪、余江为两翼，以道教文化为特色的鹰潭大旅游”格局，把鹰潭建设成中国道教文化圣地和沿海发达地区观光休闲及养生度假的“精品后花园”。力争到2010年，旅游总收入占全市生产总值比重达到15%，成为我市国民经济的重要支柱产业。加快现代商贸流通业的发展。抓好商业网点规划布局建设，在完善现有眼镜、毛发、建材、五金交电、水产品、副食品及农副产品等市场的基础上，着力发展铜产品、旅游商品、粮食、蔬菜、花生、竹木、再生资源、机动车辆等专业批发交易市场，建设鹰潭市物流中心，将我市建成赣东北货物集散地。进一步完善和优化房地产业发展，扩大经济适用房和廉租房规模。大力发展现代金融保险业和现代社区服务业。

第六，加强基础设施建设，为加快发展提供有力支撑。加快以公路为重点的交通建设。实现市至县通一级公路，县至乡通三级以上公路。加快新建鹰潭港客运码头和作业区的建设。力争赣东机场定点鹰潭并开工建设，从而形成铁路、公路、水运、航空综合运输体系，进一步强化鹰潭交通枢纽地位。强化能源保障体系。力争开工建设贵溪电厂“以大代小”改造工程，将鹰潭建成江西重要电力供应基地。加快输变电工程建设，在“十一五”期间，新建1座50万伏和一批22万伏输变电工程，扩建一批11万伏输变电工程，使全市广大地区都能享受到充足的电力保障。加强防洪保安体系建设。继续实施和完善万亩以上圩堤保护和防洪工程建设，重点抓好信江、白塔河、罗塘河等江河圩堤加固和中小型病险水库除险加固，提高防洪能力。加强森林防火体系建设，提高森林防火科学化、专业化、规范化水平。加速推进信息化建设，为建设“数字化鹰潭”奠定基础。

第七，实施科教兴市和人才强市战略，建设创新创业鹰潭。《纲要（草案）》指出，“十一五”期间要以科学发展观为指导，全面落实科技是第一生产力和人才是第一资源的战略思想，切实把科技教育放在更加重要的位置，以教育的优先发展培育造就一批创新型、创业型人才，推进自主创新，推动全民创业。加快科技创新，强化企业在自主创新中的主体地位，争取在生物医药、水表、新材料、节能光源等重点领域取得一批自主知识产权，争取在农业、资源、生态环境等领域取得一批重大科技攻关成果。优先发展教育，不断加大农村教育投入，逐步缩小城乡差距，大力发展职业教育。积极发展高等教育，整合资源，组建一所地方性本科高等院校。壮大人才队伍，提高发展竞争力。拓宽创业领域，优化创业环境，营造创业文化，培育创业主体，切实掀起全民创业的热潮。

第八，坚持可持续发展，建设生态鹰潭。积极推进产业结构战略性调整，严格控制高耗能、高污染项目建设。调整优化能源结构，实施城市管道燃气工程。大力发展循环经济，建立资源循环利用机制和城乡再生资源回收利用体系。加大环境保护力度，切实保护自然生态，做好信江、泸溪河、罗塘河、周坊河水质保护工程，严格控制废气烟尘排放。按照花园城市、园林城市和生态城市要求推进城市化进程，创建绿色居民庭院和社区，绿化、美化人居环境。

第九，关心群众生活，努力维护社会稳定。努力扩大就业和再就业门路，加大农村剩余劳动力转移力度，建立健全社会保障制度，加快公共卫生体系建设步伐。完善社会稳定保障机制，坚持依法行政、公正执法，建立健全矛盾排查机制、信息预警机制、应急处置机制，加强全社会治安综合防控体系建设，全面提高安全生产水平。

三、关于2006年国民经济和社会发展主要工作

各位代表，2006年是实施“十一五”规划的头一年，认真做好今年的各项工作，开好头，起好步，对于促进鹰潭加速崛起，顺利实现“十一五”时期各项目标任务具有十分重要的意义。

今年经济和社会发展的总体要求是：以只争朝夕的精神、开拓创新的作风、全民创业的合力，在奋力争先中求主动、在全面进步中促崛起，强势推进产业发展、城乡建设、环境升级，力求生产总值、财政收入、招商引资增幅“三个站前列”，紧扣为“十一五”开好头、奠好基的目标，努力打好提前量，坚实迈出第一步。主要预期目标是：生产总值增长13%；财政总收入增长15.3%，其中地方财政收入增长13.2%；全社会固定资产投资增长21%；居民消费价格上涨幅度控制在3%以内；城镇登记失业率控制在4.5%以内；人口自然增长率控制在7.75‰以内。要实现以上经济和社会发展预期目标，必须做好以下工作：

1、切实做好“三农”工作，加快推进社会主义新农村建设

继续高度重视粮食生产，全面贯彻落实中央和省各项支农惠农政策，粮食总产稳定在55万吨以上。严格执行国家粮食政策，切实保护农民利益。优化农业区域布局和农村经济结构，按照“品种特色化、基地规模化、生产标准化、经营产业化、投入科技化”的要求，进一步做强苗木花卉、瘦肉型猪、果业、笋竹、花生等五大优势产业，重点发展笋竹两用林、油茶、板栗、网箱养鱼、中药材，使之成为优势产业和农业新亮点。加快推进农业产业化经营步伐，发展壮大龙头企业，重点支持江西可生生化公司千吨果蔬粉体加工GMP生产线项目，贵溪红谷养鸡场30万羽蛋鸡养殖扩建、金沙蔬菜加工厂3000吨出口蔬菜加工扩建、余江大地蔬菜制品公司6000吨特色风味系列食品技改等国家、省级龙头项目建设，力争全市农业产业化龙头企业实现销售收入增长20%。抓好农村基础设施和生态建设，实施好贵溪市雷溪、塘湾、志光、泗沥、余江县画桥、月湖区童家、龙虎山景区上清等乡镇国家投资总面积6.8万亩的土地整理及园田化项目，力争余江10万亩农田列入全国基本农田保护示范区。做好白塔渠水利血防巩固工程、中潢圩堤和病险水库除险加固、机电泵站改造以及农村饮水安全等一批水利工程建设。继续实施好国债长防林、农发长防林、生态公益林、退耕还林等国家林业重点工程，组织实施森林重点火险区综合治理项目。完成鹰东连接线通道绿化工程。继续加大农村通村水泥路建设。扎实推进社会主义新农村建设。认真落实工业反哺农业、城市支持农村的方针，坚持多予、少取、放活，使农业生产力水平有一个较大提高，广大农民的生活有明显的改善，农村基础设施建设得到切实加强，农村各项社会事业全面发展，农村基层民主建设继续推进。

2、加快新型工业化步伐，不断提升工业化水平

做大做强主导产业，紧扣铜材加工、眼镜加工、医药化工、微型元件、食品加工等特色产业，延伸产业链，不断扩大产业规模和竞争能力。支持重点企业加快发展，倾力支持江铜集团公司加快30万吨铜冶炼和铜精深加工项目建设，支持贵电、贵化、三川水表、南方照明、诚志生化、海燕实业、科华水泥、海利农药、耐乐铜业等重点企业加快发展，促其提速增效。推进工业园区土地集约化经营、产业链式化延伸、资源循环式利用，提高工业园区单位面积投入强度和产出水平。市工业园区要继续完善基础设施建设，改造110千伏鹰余输电线路，建成110千伏变电站，进一步完善供电、供水网络，确保入园企业合理用地、用电、用水。加快创新扶持体系建设，鼓励企业自主创新。注重发展循环经济，推动企业安全生产、清洁生产、节约生产。建立健全工业经济监测和协调机制，更加注重加强煤电油的区域性、季度性调度，更加注重抓好银企合作沟通，为工业生产提供良好发展环境。

3、大力发展旅游、流通业，不断壮大第三产业

积极实施品牌带动战略，充分发挥“中国优秀旅游城市”的品牌效应，大力开展创建“全国文明景区先进单位”、“中国最受外国人欢迎的景区”活动，扎实做好创全国“5A”级旅游区的前期准备工作，稳步推进龙虎山申报世界地质公园、世界自然和文化遗产以及上清古镇申报全国历史文化名镇工作。积极申报江铜工业游、白鹤湖农业生态游为全国工业、农业旅游示范点。切实完善鹰潭市旅游产业总体规划，着手编制全市“十一五”旅游发展规划以及市区、贵溪、余江旅游产业发展规划，高标准完成龙虎山风景名胜区总体规划修编。全面启动龙虎山道教博览中心和上清古镇开发建设，加快天鹅湖景点、上清旅游公路等旅游基础设施建设。创新旅游宣传促销手段，加强在中央电视台等主流媒体和周边重点客源市场多种媒体的宣传，大力开展节庆促销、区域联合促销，与中国道教协会、中国旅游协会联合举办中国道教名山旅游合作大会。加快旅游专列引进步伐，全年确保引进旅游专列10趟以上。积极拓展海外客源市场，成立一家国际旅行社，争取与韩国等国家的重点旅游客源城市结为友好城市。加强旅游行业管理，强化从业人员教育培训，规范旅游市场秩序，全面提升管理和服务水平。全市实现接待国内旅游者增长14.4%，接待境外旅游者增长20%，旅游总收入增长19%，旅游创汇增长21%。

充分发挥区位和交通优势，大力发展物流业、商贸业、现代运输业和住宿餐饮业等现代服务业。加快鹰潭物流中心和专业市场建设步伐，引进大型超市和商贸企业，积极培育现代社区服务业和信息、会计、咨询、法律等中介服务业。加大对食品、药品、农资、建材等商品为重点的专项打假活动，进一步规范市场经济秩序。

4、着力推进新型城镇化，不断提高城市发展水平

巩固创建中国优秀旅游城市、省级卫生城市成果，继续开展创建全国双拥模范城市和省级园林城市活动。坚持高标准规划。开展《鹰潭市主城区2006年至2010年近期建设规划》和《夏埠区控制性详细规划》编制工作。坚持高质量建设。启动雄鹰大道、龙虎山大道延伸段等一批城市道路建设工程。继续做好320、206国道配套改造项目建设。加快胜利文化休闲广场、东湖公园二期、沿江景观、管道燃气工程建设。完成污水处理一期、江南水厂扩建、鹰潭公园改造工程。支持配合景鹰高速公路（鹰潭段）建设，加快景鹰高速公路鹰潭连接线、贵溪入闽公路、应天山至大脚岭旅游道路工程、市县至乡国债公路等一批基础工程建设。加快市体育馆、市人民医院门诊医技大楼建设。继续深入开展“告别陋习，树立新风”活动，促进城市“软件”与“硬件”协调发展，努力把我市建设成为文明、整洁、安全、有序的新兴旅游城市。

5、进一步深化改革开放，不断增强经济发展的活力与后劲

加快推进以乡镇机构、农村义务教育和县乡财政体制改革为主要内容的农村综合改革。深化财税体制改革。完善国有资产监督管理体系，妥善处理国有企业改制遗留问题。启动市政公用事业改革，不断扩大事业单位聘用制推广面。深化粮食流通体制改革、林业产权制度改革和水利产权制度改革。大力推进投融资体制改革，不断规范投资备案制和核准制。

坚定不移地实施大开放主战略，以开展好“安商亲商服务年”活动为抓手，以工业项目招商和安商亲商服务为重点，着力引大引强，形成产业集聚和成龙配套的发展态势。紧扣产业发展主线，充分发挥区位、交通、产业特色和服务优势，发挥“浙商最佳投资城市”品牌优势，努力在引进内资超亿元工业项目和外资超千万美元项目上形成新突破。实行领导和部门联系客商、客商评议评价环境、涉企检查告知等制度，强化安商责任，让客商在鹰潭真正有“家”的感觉，使鹰潭成为投资者的第二故乡。转变对外贸易增长方式，不断培植出口大户和新户，提高出口创汇能力。实际引进市外资金26亿元，其中引进省外5000万元以上工业项目资金23亿元，实际利用外资5600万美元，外贸出口4000万美元。

6、大力发展各项社会事业，努力构建和谐平安鹰潭

按照科技进步示范市建设规划要求，围绕新材料产业化，尤其是铜材加工产业化，组织申报国家级科技项目6项、省级科技项目15项。继续推进与高等院校、科研机构的合作与交流，增强自主创新能力。进一步推进农村科技特派员工作，加大知识产权保护力度。完善以县为主的教育管理体制。进一步巩固九年制义务教育成果，争取更多资金用于改善农村中小学办学条件、对家庭困难学生实行“两免一补”和发展农村远程教育。继续推进优质高中建设，加快发展高等教育、职业教育和民办教育。改善医疗卫生条件，进一步加强重大疾病防治工作。加大乡镇卫生院改造力度，支持贵溪市、余江县开展新型农村合作医疗试点改革，巩固血防成果。强化食品药品安全监管，保证人民群众饮食用药安全。采取有效措施，继续稳定低生育水平，综合治理出生人口性别比失调问题。加强文物保护工作和文化宣传基础设施建设，努力创作文艺精品。加快广播电视“村村通”工程建设步伐，扩大农村广播电视覆盖范围。精心组团参加省十二届运动会，积极申办2010年省十三届运动会。不断完善人才资源开发管理体制和运行机制，构筑人才聚集高地。继续强化社会治安防控体系建设，开展多种形式的严打专项整治活动。制定并启动“五五”普法规划，进一步加强矛盾纠纷调处和法律援助工作。认真落实信访条例，依法认真做好信访工作。抓住安全监管不放松，深入开展隐患排查和整治工作，确保安全生产态势平稳。

7、高度关心群众生活，努力解决群众实际困难

坚持就业优先原则，采取政策扶持与市场择业相结合的办法，建立健全与经济发展水平相适应的社会就业服务体系。妥善解决高校毕业生、失地农民的就业问题，重视做好退役军人和随军家属的就业安置工作。力争全年新增城镇就业1.8万人，下岗失业人员再就业6000人，困难人员再就业1200人，城镇登记失业率控制在4.5%以内。大力开展职业技术培训，提高就业和再就业者素质。加快完善社会保障体系，改革基本养老金计发办法，按照省政府统一部署，逐步做实企业职工基本养老保险个人帐户。有效开展社会养老保险、失业保险扩面征缴工作，大力推进城镇基本医疗保险制度改革，引导困难企业和职工、下岗失业人员和就业困难人员参加不同层次的医疗保险。提高城市最低生活保障补助标准，开展农村最低生活保障制度试点，加快新型社会救助体系建设。继续加大农村敬老院、光荣院、福利院基础设施建设力度，提高农村“五保”集中供养率。进一步落实减轻农民负担、增加农民收入的政策措施，切实提高城镇居民尤其是中低收入者的收入水平。

8、切实转变政府职能，不断提高政府工作水平和效能

建设法治型政府。全面推进依法行政，各级政府都要按照法定权限和程序行使权力、履行职责。政府工作人员要学会并善于依法处理经济社会事务。改革行政执法体制，相对集中行政许可权和行政处罚权，推进综合执法试点，解决多头执法等问题。加强行政执法监督，促进严格执法、公正执法和文明执法。实行执法责任制和执法过错追究制，完善并严格执行行政赔偿制度。各级政府要自觉接受人民代表大会及其常委会的依法监督和人民政协的民主监督，认真听取民主党派、工商联、无党派人士和各人民团体的意见，及时办理人大代表议案、建议和政协委员提案。

建设服务型政府。各级政府工作人员，要切实改进工作作风，提高办事效率和办事水平。要牢记党的宗旨，经常深入基层调查研究，倾听群众呼声，关心群众疾苦，帮助群众解决实际困难。要增强破解难题的能力，善于发现问题，解决问题。要增强责任感和事业心，忠于职守、勤奋敬业，切实做好本职工作。

建设廉洁型政府。大力推进惩治和预防腐败体系建设，从源头上预防和治理腐败。加强监督检查，以解决群众反映的突出问题为重点，加大专项治理力度。加强行风建设，坚决纠正损害群众利益的不正之风。严格执行领导干部廉洁从政各项规定，坚决查处违反政纪的案件。加快推进有利于反腐倡廉的机制体制建设，逐步铲除腐败现象滋生蔓延的土壤和条件。

各位代表，“十一五”时期是全面建设小康社会的关键时期，也是我市改革发展进程中的一个重要时期，“十一五”期间的任务光荣而艰巨。让我们紧密团结在以胡锦涛同志为总书记的党中央周围，高举邓小平理论和“三个代表”重要思想伟大旗帜，全面贯彻落实科学发展观，团结奋进、开拓创新，为加速鹰潭崛起，实现小市有大作为而努力奋斗！
